# Detection of residual cognitive function through non-spontaneous eye movement in a patient with advanced frontotemporal dementia

**DOI:** 10.3389/fnins.2014.00334

**Published:** 2014-10-24

**Authors:** Akira Midorikawa, Chihiro Itoi, Mitsuru Kawamura

**Affiliations:** ^1^Department of Psychology, Chuo UniversityTokyo, Japan; ^2^Department of Neurology, School of Medicine, Showa UniversityTokyo, Japan; ^3^Department of Psychology, Chuo University Graduate SchoolTokyo, Japan

**Keywords:** dementia, frontotemporal dementia, frontotemporal lobar degeneration, eye movement, saccadic reaction time

## Abstract

As dementia progresses, cognitive functions decline in patients, and caregivers and other support staff gradually lose the means to communicate with them. However, some caregivers believe that patients can still recognize their surroundings even when they show akinesis with mutism. In this study, we observed eye movements (preferential-looking paradigm) to detect the presence of residual cognitive functions in a patient with severe frontotemporal dementia (FTD). The subject was a 76-year-old female. At the time of observation, she had lost all spontaneous activities. Magnetic resonance imaging (MRI) imaging showed dense atrophy of the bilateral frontotemporal lobe, but the parieto-occipital lobe was preserved. A preferential-looking paradigm was used in the experiment, whereby two different faces (learned and non-learned) were simultaneously presented to the patient on a TV monitor. As a result, we found no significant differences in looking time between the two faces. However, when the saccade timing to the presented faces was examined, a much longer latency was observed for the right than the left side of the target faces. Even though the patient had lost capacity for spontaneous activity due to severe FTD, we were able to observe partial residual cognitive ability using the eye-movement paradigm.

## Introduction

As dementia progresses, patients' cognitive functioning declines, and caregivers and other support staff gradually lose the means to communicate with them. However, some caregivers believe that patients can still recognize their surroundings even when they show akinesis with mutism. In this study, we employed a preferential-looking paradigm, observing the eye movements of a patient with advanced frontotemporal dementia (FTD) to detect any residual cognitive functions. We aimed especially to study facial recognition ability in a patient with advanced FTD who did not show any spontaneous activity, including eye movement, but showed preserved exploratory eye movement for faces. Because face preference is shown in early infancy (Morton and Johnson, 1991), we hypothesize that face preference is a primitive function, and that this function is preserved even in advanced FTD patients.

## Background

### Case history

**Figure 1:**
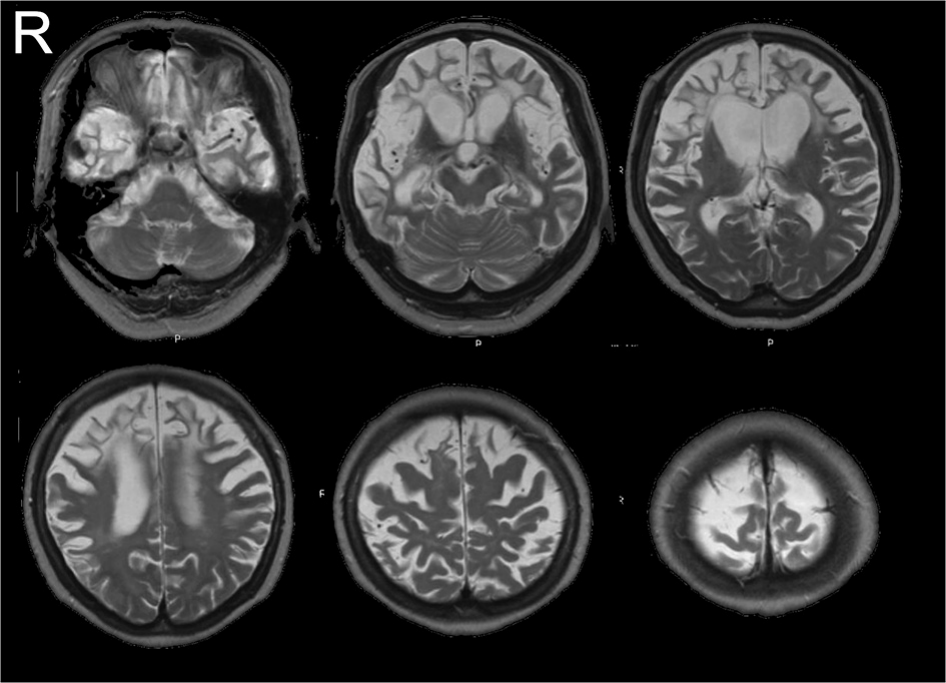
**MR images (July 2006)**. MRI revealed dense atrophy of the bilateral anterior (frontotemporal) lobule, but the posterior (parieto-occipital) lobule was well-preserved.

In December 2002, a 66-year-old female patient was admitted to the Department of Neurology, Showa University Hospital, in Tokyo, Japan, after she presented with severe word-finding difficulty and a substantial change in personality. Her symptoms had begun at the end of 2000, and her neurological examination (conducted at another hospital) was normal, with the exception of verbal impairment. Her spontaneous speech had decreased, but she was capable of shopping and preparing a meal by herself. Over the next 2 years, her condition markedly deteriorated. She was unable to perform most spontaneous activities but was still able to follow instructions, walk, and eat independently. In February 2003, her score on the mini-mental state examination (MMSE) (Folstein et al., 1975) was 1 point (only the “repetition” test was correct), and her Mental Function Impairment Scale (MENFIS) (Homma et al., 1991) score (72/78) showed severe deterioration of several functions, with the exception of topographical disorientation. At that time, neurological examination showed a strong grasping reflex of both the hands of the patient (the left hand more so than the right hand) and compulsive laughter. Three years later (November 2006), due to severe cognitive decline, we could not perform any neuropsychological examinations, including MMSE. In daily situations, she was unable to perform most spontaneous activities, including speaking, but could still perform some activities, such as following instructions (e.g., “catch the ball”), eat with chopsticks, and skillfully cut paper with scissors even though she showed a grasping reflex (Midorikawa and Kawamura, 2010). In July 2006, magnetic resonance imaging (MRI) revealed dense atrophy of the bilateral anterior (frontotemporal) lobule, but the posterior (parieto-occipital) lobule was well-preserved (Figure 1). Single photon emission computed tomography (SPECT) imaging also showed bilateral frontotemporal hypoperfusion (the right side more than the left side). During this period, the patient met the criteria for clinical diagnosis of FTD (Neary et al., 1998) or probable behavioral variant frontotemporal dementia (probable bvFTD) (Rascovsky et al., 2011).

In June 2007 (5 years after her initial examination), the patient was still awake during the daytime but was unable to follow instructions. Thus, we could not examine her cognitive abilities because of her severe cognitive decline. However, she was still able to navigate certain areas of her neighborhood with her husband. Not only was she able to take a walk without losing her way but also she could take a different route every day. She particularly preferred the narrow back streets rather than the main street. She also could have a meal independently using a pair of chopsticks skillfully, but she picked up tiny residual food particles in the bowl over and over again. In November 2011 (9 years after her initial examination), with the exception of a few hours each day when her husband brought her to the living room to eat meals, she could no longer walk around and spent most of her days and nights in bed or seated in front of the television. Because our patient had no spontaneous eye movement under instruction but sometimes seemed to be showing exploratory and pursuit eye movement when she looked at a real moving face, we hypothesize that she still was able to discriminate people in her surroundings. To explore her residual cognitive ability, we adopted the preferential-looking paradigm for face stimuli. Before the experiment, verbal informed consent was obtained from the patient's caregiver. (Because our college has no established ethical committee, we could not obtain ethical review approval.)

### Experimental setting

**Figure 2:**
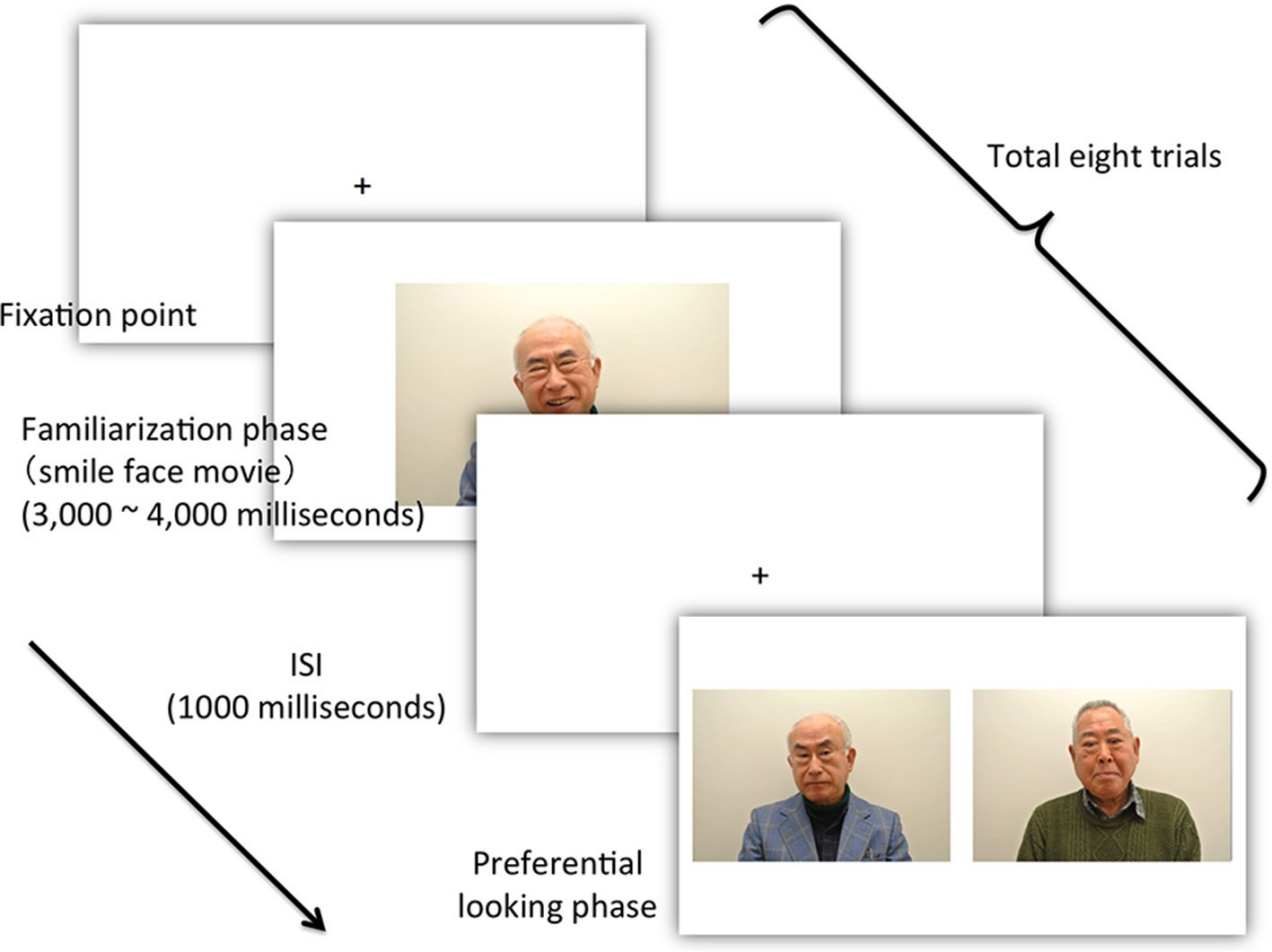
**The flow of the experiment**. After presentation of the fixation point and an accompanying sound signal, the experimenter confirmed that the patient looked at the TV monitor and then pressed the start key, which initiated the presentation of the movie (Actual photos were taken from young male actors.).

The patient was first familiarized with a movie of a smiling face. The familiarization phase was fixed at a relatively short duration (3000–4000 s). After familiarization, the patient was tested using a pair of faces, one novel and one familiar (Figure 2). To evaluate face discrimination ability, we used a preferential-looking paradigm. Because the preferential-looking paradigm was non-invasive and the patient required no understanding of the paradigm's intention, we believe it was appropriate for people who could not communicate because of advanced dementia. In addition, because moving facial expression facilitates infants' face recognition (Otsuka et al., 2005), we hypothesize that it might be the primitive function that is functional during advanced dementia. Therefore, we adopted a movie of smiling faces as a familiarization stimulus. The patient was seated in a chair located approximately 1 m from a 32-inch TV monitor (REGZA 32H1, Toshiba Corporation, Tokyo, Japan). No specific instructions were given as the patient had an established habit of watching TV every day. Although there was no guarantee that the patient was actually watching the TV monitor, sitting in front of the TV was one of her usual activities: her eye movements were observed, and they corresponded to activity on the TV. The TV monitor was connected to a laptop (VPCZ12, SONY, Tokyo, Japan) via HDMI and controlled by it. A compact video camera (DCR-PC300, SONY Japan), placed in front of the patient under the TV monitor, recorded her eye movements in infrared mode.

### Stimuli

All stimuli were produced from a video recording of unfamiliar young male Japanese faces. In the familiarization phase, the stimuli consisted of a short movie of smiling young male faces. In the preferential looking phase, 16 photographs were used as the stimuli. Eight of the 16 photographs were used as targets (presented in the familiarization phase), and the remaining eight photographs were used as distractors. Each photograph in the preferential looking phase was selected from movie footage when the actors' facial expressions were emotionally neutral. The familiarization movies' duration ranged from 3000 to 4000 ms, corresponding to duration of the facial expression.

The experiment was managed using computer software (SuperLab 4.5, Cedrus Corporation, CA, USA). Eight trials were performed. Figure 2 shows the time schedule for the experiment. The computer emitted a sound to signal the beginning of each trial. The experimenter then confirmed that the patient was looking at the TV monitor, and pressed the start key to begin presentation of the movies (familiarization stimuli). The resolution of each movie was 720 × 480 pixels. Each movie depicted a smiling facial expression lasting between 3000 and 4000 ms. After a 1000-ms-interval (inter-stimulus interval: ISI), two simultaneous still pictures were presented until the patient moved her gaze from the TV monitor. The two simultaneous pictures comprised a learned familiarization face (target stimulus) (720 × 480 pixels) and an unfamiliar, non-learned face (distractor stimulus). The size of the screen was 1600 × 900 pixels. The size of each picture was identical to that of the movie (720 × 480 pixels), and the distance between the two was 80 pixels. The position of the target stimuli in the eight trials was counterbalanced across trials. During the experiment, we recorded and observed the patient's eye movements using the video camera recorder.

### Data analysis

Following the presentation of the simultaneous pictures, saccades from the center position and total looking time for each photo were evaluated using videotape of the patient's eye movements. Looking times for both target stimuli and distractors, and saccade latencies for both targets and distractors were analyzed. The frame rate of the video camera was 30 fps. Thus, the accuracy of the analysis was 1/30 s (33.3 ms). Statistical analysis (Welch's *t*-test) was performed using the R (R Core Team, 2013). The significance level was set at *p* < 0.05.

### Results

**Figure 3:**
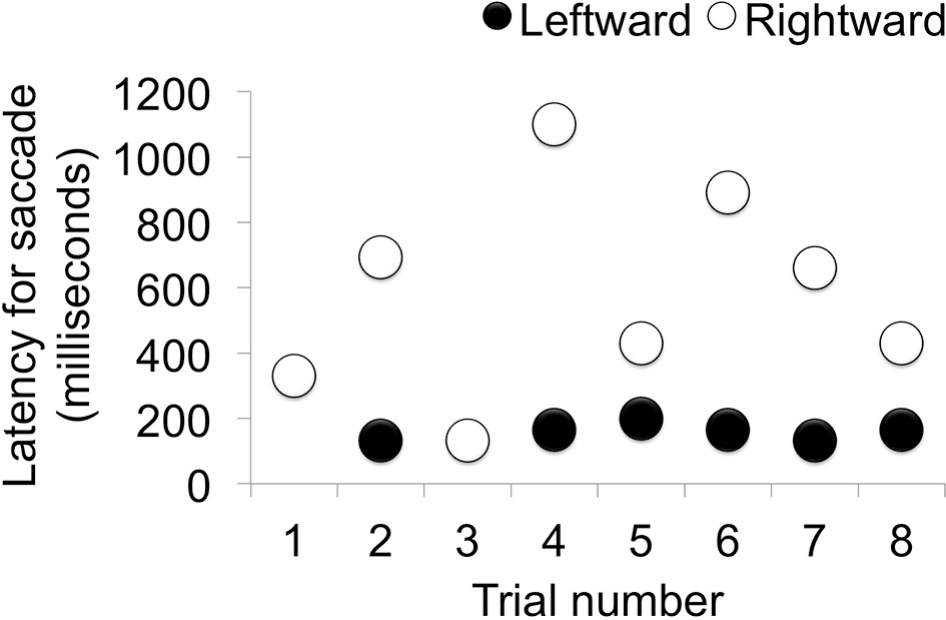
**Saccade latency in each trial**. When the latency of the saccade was analyzed, left-side latency was stable and right-side latency varied depending on the condition (Trials 1, 3, 5, and 8 were conducted under the same conditions.).

**Figure 4:**
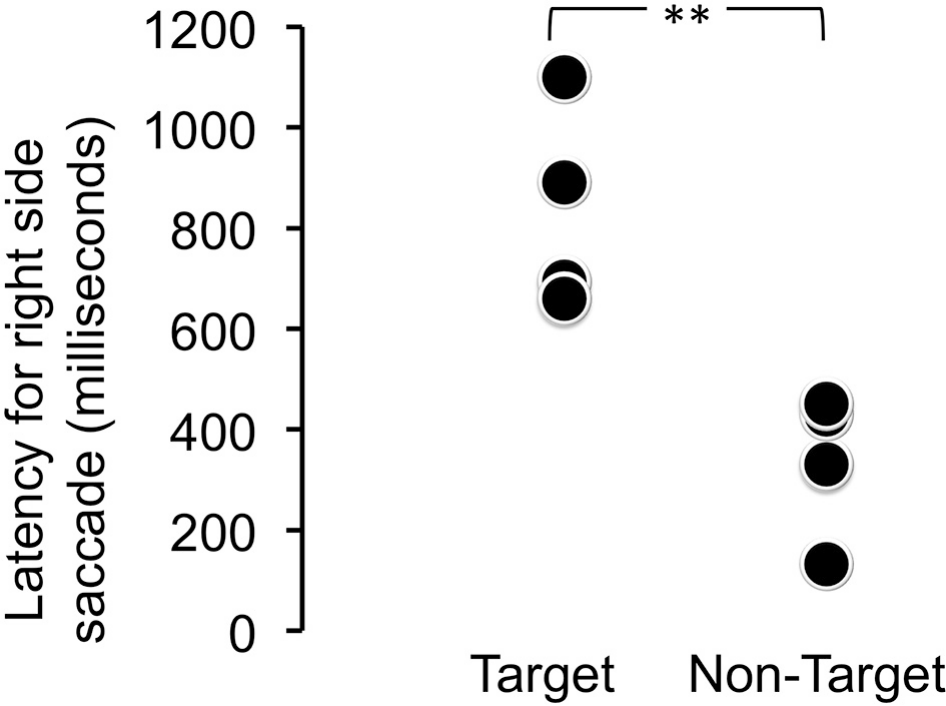
**Latency for the right-side saccade for target (learned) and non-target (novel) face stimuli**. The latency for the right side was significantly delayed when the patient looked at the learned face [*t*_(6)_ = 4.1019, ^**^p < 0.01].

When viewing time and direction were analyzed, statistically significant differences were not found between target and distractor stimuli [*t*_(9.68)_ = 0.0514, *p* = 0.9601] (Table 1), but looking times were biased toward the right side (Table 2). Analysis of the saccade latencies revealed significant differences between right and left (Figure 3). The left saccades were observed in six of the eight trials and were stable (159.5 ± 22.6 ms). The right saccades were observed in all eight trials, and the latencies varied (582.8 ± 294.5 ms). In addition to the latency analysis, when the type of the stimuli was considered, an apparent discrepancy was observed. When the patient looked at the learned face, the latency for the right side was significantly delayed [*t*_(6)_ = 4.1019, *p* < 0.01] (Figure 4).

**Table 1:** **Looking time for target and non-target stimuli**.

	**Average looking time (ms)**	**Ratio (%)**
Target	4705.0	50.8
Non-target	4549.5	49.2

**Table 2:** **Looking time for right and left stimuli**.

	**Average looking time (ms)**	**Ratio (%)**
Right	7624.1	91.8
Left	445.5	8.1

## Discussion

In the course of the disease, as typical of bvFTD patients, our patient showed dense frontotemporal atrophy and a relatively preserved posterior cortex. Nevertheless, until the middle stage of the disease, she could walk around without losing her way, and she showed good visuospatial functions (good skill with scissors) (Midorikawa and Kawamura, 2010). However, when her disease progressed, she did not show any preserved ability due to her severe state of akinesis with mutism. Because the posterior part of her brain was relatively preserved and she showed some eye movement when she was in front of the TV or when her caregiver was face-to-face. Also, sometimes she looked like she was comparing two persons' faces with her eye. Therefore, we could postulate that, even though we had had no evidence of her internal or latent of cognitive function, her residual functions of the posterior cortex had been preserved, but until now, we had no guarantee that she could discriminate between two persons, and there were no methods to detect her residual cognitive ability.

In this study, our initial aim was to use a preferential-looking paradigm to establish whether any internal or latent cognitive functions could be detected in a patient with severely advanced bvFTD. The preferential-looking paradigm was chosen because it is non-invasive, and the subject does not need to learn how to behave with regard to either the stimuli or the experimental setting or to show any spontaneous activity or intention. The preferential-looking paradigm has previously been established as suitable and valid for evaluating cognitive ability in subjects such as non-experimental primates located in restricted environmental situations (Hanazuka et al., 2012) or human subjects with limited cognitive abilities (Woodhouse et al., 2007). Therefore, we were confident that the preferential-looking paradigm was appropriate for testing the cognitive capacity of the patient with advanced FTD.

Although our experimental design yielded a negative result to identify any significant difference between familiar target (learned faces) and non-target stimuli (distractor faces), significant side bias was observed. Our analysis of the latency periods for the first saccades revealed an apparent difference between target and non-target stimuli. Therefore, in addition to looking time and gaze direction, saccade latency was analyzed, and the results suggested residual internal cognitive ability despite the subject's severe dementia and incapacity for spontaneous activity.

Few studies report findings obtained using methods that compensate for the communication disabilities of patients with advanced dementia. In fact, to our knowledge, only a few trials based on a classical conditioning paradigm, have been published (Liberati et al., 2012). However, this report was hypothetical, and the validity of the paradigm for patients with dementia was not determined. Recently, the same protocol was applied to patients (Liberati et al., 2013), but the subjects had mild Alzheimer's. A similar study, conducted by Woodhouse et al. (2000), used a preferential-looking paradigm to examine the visual acuity of subjects with learning disabilities. However, this study assessed only visual acuity. Therefore, our application of a preferential-looking paradigm to determine latent cognitive capabilities of a patient with advanced dementia is unique.

Previous research has shown that the eye-movement paradigm not only detects attention or cognitive function, but can enable communication with, or further evaluation of, patients with amyotrophic lateral sclerosis (Evdokimidis et al., 2002) or other neurological disorders (Tobii Technology)^1^. Although, here, the use of this paradigm indicated residual cognitive functions, it did not facilitate communication because we could not confirm whether our patient's eye movements were caused by conscious intent or by spontaneous mental activity.

In terms of the neural substrate underlying our paradigm, it has been reported that face identification takes place in the ventral temporal cortex (Haxby et al., 2001). Our patient's imaging results fit well with these observations, as her posterior cortex, including the temporal, parietal, and occipital cortices, was well-preserved. Additionally, a direct pathway of saccade eye movements has been reported between the temporal cortex and basal ganglia (Schiller and Tehovnik, 2005). Therefore, we hypothesize that our patient's different saccadic patterns for known and unknown faces was residual behavior. This might be due to an intact neural “reflex” unaffected by the dementia.

Our study had several limitations. First, it was based on a single subject. Therefore, we cannot conclude that our adopted method has broad utility for patients with dementia. Second, our experiment consisted of merely eight trials. Our results showed clear differences between target and non-target stimuli, but we cannot completely reject the statistical errors. Nevertheless, we think that this adopted method has the potential to be a window for patients with dementia, especially advanced FTD. First, the preferential-looking paradigm is a non-invasive method, demanding nothing from the subject. Therefore, we believe this method is suitable for people with low ability. Second, our patient had no spontaneous eye movement, but could discriminate familiar and unfamiliar faces using a first look latency; this means that looking time and latency are crucial for people with low ability.

Although our patient could follow the movements of familiar faces and scan the TV monitor prior to the experiment, the significance of her eye movements was not clear. Were they a neurological reflex or were they meaningful behavior? Our finding offers hope for patient caregivers and families with regard to determining the remaining cognitive function of patients with advanced FTD.

## Concluding remarks

Even though the patient had lost capacity for spontaneous activity due to FTD, we were able to observe partial residual cognitive ability using the eye-movement paradigm.

### Conflict of interest statement

The authors declare that the research was conducted in the absence of any commercial or financial relationships that could be construed as a potential conflict of interest.

## Footnotes

^1^Available at: http://www.tobii.com/en/assistive-technology/global/disabilities/
